# Evaluation of Eye Irritation Potential of Aqueous Leaf Extract of *Achyranthes aspera* by *In Vitro* and *In Vivo* Method

**DOI:** 10.5402/2012/693489

**Published:** 2012-01-31

**Authors:** Gajanan Rajpal Deshmukh, Kuntrapakam Hema Kumar, Poojari Venkata Suresh Reddy, Boddapati Srinivasa Rao, Chirumamilla Venkata Satish Kumar

**Affiliations:** ^1^Toxicology, Aptus Biosciences Private Limited, SVS Medical College Campus, Yenugonda, Andhra Pradesh, Mahabubnagar 509 002, India; ^2^Quality Assurance Unit, Aptus Biosciences Private Limited, SVS Medical College Campus, Yenugonda, Mahabubnagar 509 002, India

## Abstract

The present paper is an attempt to investigate the eye irritation potential of aqueous leaf extract of *Achyranthes aspera* by *in vitro*, Hen's Egg Chorioallantoic Membrane Test (HET-CAM) and *in vivo* acute eye irritation test in rabbits. The irritation score (IS) obtained after treatment of the extract on HET-CAM is 0.07 and that of in rabbits is 0.55, Which does not comes under either category 1 or 2 as per the harmonized integrated classification system. The aqueous extract of *Achyranthes aspera* showed no eye irritation properties both *in vitro* and *in vivo* methods when compared with negative control whereas positive controls showed eye irritation potential.

## 1. Introduction

The eyes are one of the most delicate parts of our body. They can be exposed to cosmetic products and their ingredients through either use of products directly (mascaras, eye creams) or accidentally (which may enter the eye). The evaluation of eye irritation potential for a cosmetic product and its ingredients is essential to provide reassurance that a product is safe for consumers to use through intended and foreseeable uses and accidental exposures to the eye.

The conventional method for determination of the irritant or corrosive potential of chemicals is an acute eye irritation test and has become the international standard assay for acute eye irritation and corrosion. This test involves an examination of cornea, conjunctiva, and iris for three days after application of test item to the one eye of a rabbit [1].

An extensive list of *in vitro* models have been developed and proposed as an alternative to the acute eye irritation test [2–4]. Although some of the many alternative assays developed have received limited attention, substantial efforts have been investing in evaluating a significant number of the assays. One of the alternative *in vitro* methods is a Hen's Egg Chorioallantoic Membrane Test (HET-CAM).

*Achyranthes aspera *(Amaranthaceae) is an important medicinal herb found as a weed throughout India. Though almost all of its parts used in traditional systems of medicines, seeds, roots, and shoots are the most important parts, which are used by traditional healers for the treatment of fever, dysentery, and diabetes [5–7]. The present paper is an attempt to evaluate the eye irritation potential of aqueous leaf extract of *Achyranthes aspera* by *in vitro*, Hen's Egg Test—Hen's Egg Chorioallantoic Membrane Test (HET-CAM) and *in vivo* acute eye irritation test. According to OECD test guideline 405, the investigation of acute eye irritation test in rabbits was carried out.

## 2. Materials and Methods

### 2.1. Plant Material and Preparation of Leaf Aqueous Extracts

The fresh leaves of *Achyranthes aspera* were collected during January–March in and around the Yenugonda village (Mahabubnagar district, Andhra Pradesh, India.). The leaves were cleaned with distilled water and shade-dried at room temperature. The dried leaves were powdered (100 g) and were aqueous-extracted separately to exhaustion in a soxhlet apparatus using the aqueous solvent system. The aqueous extract is filtered through Whatman filter paper no. 1 and then concentrated by evaporating at low temperature (40–50°C) to get 3.16 g yield from aqueous fractions. The aqueous extract is preserved in airtight containers at 4 ± 2°C until further use.

### 2.2. Test Systems

#### 2.2.1. Hen's Egg Test Choroiallantoic Membrane

The HET-CAM bioassay was performed following ICCVAM recommendations published in November 2006 in Appendix G and adapted to our laboratory conditions [8]. The fresh, clean, fertile chicken eggs weighing 40 to 50 grams are obtained from commercial sources. These eggs are candled to detect the viability and development of embryo's prior to use and nonviable or defective eggs are discarded. Finally, three eggs per group are used in the study.

**Negative Control**

A 0.3 mL of 0.9% NaCl solution is directly applied on the choroiallantoic membrane to provide a baseline for the assay endpoints and to ensure that the assay conditions do not inappropriately result in an irritant response.

**Positive Control**

0.3 mL of 1% SDS and 0.1 N NaOH are applied on the choroiallantoic membrane; a severe response in HET-CAM is expected.

**Treatment**

0.3 mL of *Achyranthes aspera *aqueous extract is applied on the choroiallantoic membrane on the 9th day. Effects are assessed in the near surroundings of the test item within 5 minutes. The time point is noted when one of the following effects occurs: hemorrhage, lysis, and coagulation. An irritation score (IS) is calculated, and the test item is classified with this score. The following formula is used to generate an irritation score (IS):

(1) $$\begin{array}{cc} \text{IS} & =\frac{\left(301-\text{Hemorrhage}\right)}{300}\times 5+\frac{\left(301-\text{Lysis}\right)}{300}\times 7\\ & \;+\frac{\left(301-\;\text{Coagulation}\right)}{300}\;\times 9, \end{array}$$

where hemorrhage is the time taken to start (in seconds) of hemorrhage reactions on CAM; lysis is the time taken to start (in seconds) of vessel lysis on CAM; coagulation is the time taken to start (in seconds) of coagulation formation on CAM.

After the treatment, the main reaction was scored within 5 minutes of time (either hemorrhage or lysis, or coagulation) according to the following scheme: 0 = no reaction; 1 = slight reaction; 2 = moderate reaction; 3 = severe reaction, and mean irritation score was determined [9].

#### 2.2.2. Acute Eye Irritation Test

The healthy young New Zealand white rabbits are used for the study with prior examination of both eyes of each experimental animal 24 hours before starting the experiment, to avoid any animals showing ocular defects or preexisting corneal injury.

**Positive Control**

0.1 mL of 1% SDS is applied on the conjunctival sac of the left eye of a single male rabbit, which is expected to induce a severe response.

**Treatment**

The test is carried out by applying 0.1 mL containing 100 mg of *Achyranthes aspera* aqueous extract in the conjunctival sac of one eye of three rabbits after gently pulling the lower lid away from the eyeball. The lids are then gently held together for about one second in order to prevent loss of the material. The other eye, which remains untreated, serves as a control.

The eyes were examined at 1, 24, 48, and 72 hours after application. The grades of ocular reaction, that is, conjunctivae, cornea, and the iris, are recorded at each examination as per OECD TG405.

## 3. Results and Discussion

**Figure 1:**
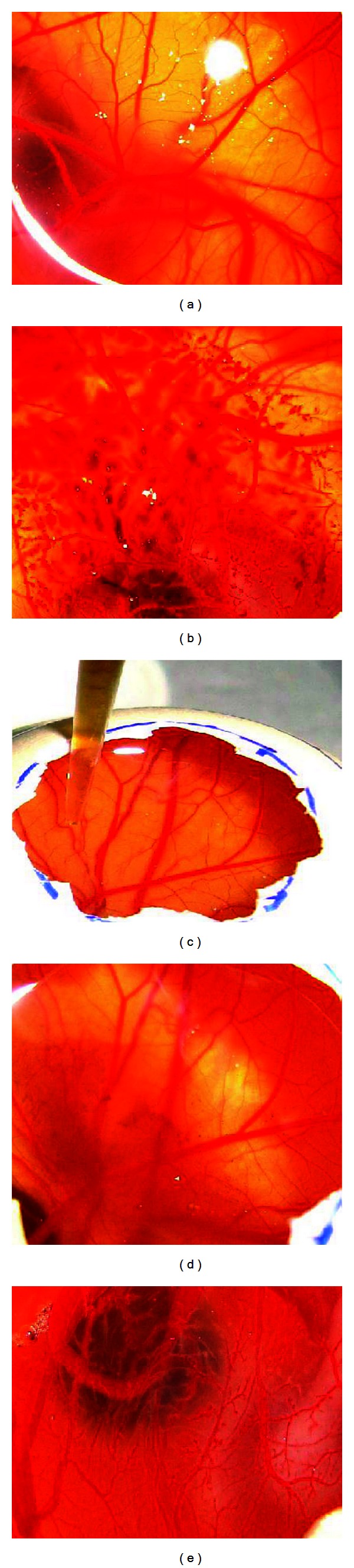
(a) Normal, (b) CAM treated with 0.1 N NaOH, (c) Treatment of CAM with extract, (d) Extract treated CAM, (e) CAM treated with 1% SDS.

The experimental results regarding the eye irritation potential of aqueous extract of* Achyranthes aspera *by *in vitro* method, that is, Hen's Egg Test Choroiallantoic Membrane (HET-CAM), is presented in Table 1 and Figure 1, while by *in vivo* method, that is, the Acute Eye Irritation test, is presented in Table 2.

### 3.1. HET-CAM Assay

The effects induced by the test compound as well as the selected controls were registered as macrophotographs representing the surface of the chorioallantoic membranes before and after treatment for 5 minutes of contact (Figure 1). 

The results show a great difference between the positive controls (SDS and NaOH) and the test compound aqueous extract of* Achyranthes aspera*. NaOH and SDS induced major damage at the vascular level of the chorioallantoic membrane (Figure 1). After the application of 0.3 ml of SDS and NaOH solution, a medium to large area was affected by the formation of microhemarrhages. The other end points noted were lysis of immature blood vessels which are the main target, finally results in degradation of blood vessels. A few areas of microcoagulation for SDS and major areas for NaOH were noted. Lastly the death of the specimen was registered after 25 and 12 minutes of contact with these solutions, respectively. On the other hand, application of 0.3 mL of aqueous extract of *Achyranthes aspera *showed absolutely no effect of hemorrhage, lysis, or coagulation or not even the death of the subject after 2 hours of observation (Figure 1(d)). 

### 3.2. Acute Eye Irritation Test

The induced effects after the single application of test compound as well as the selected control was noted by scoring the lesions of the conjunctiva, cornea, and iris, at specific intervals.

The mean score was calculated across three scoring times (24, 48, and 72 hours after treatment) for each animal for corneal opacity, iris, conjunctivae, and chemosis. The obtained numerical scores were compared with the Harmonised Integrated Classification System for Human Health and Environmental Hazards of Chemical Substances and Mixtures, 14 August, 2001 [10] (Table 2).

The acute eye irritation test in rabbits was investigated according to OECD test guideline 405. The test item, approximately 0.1 mL (containing 100 mg), was applied into the conjunctival sac of the left eye of a single male rabbit. As no severe eye, reactions were observed up to 72 hours posttreatment, the confirmatory test was followed using the remaining two male rabbits.

The scoring of eye reactions was performed at 1, 24, 48, and 72 hours for all the three animals after test item application. The mean score was calculated across 3 scoring times (24, 48, and 72 hours after treatment) for each animal for corneal opacity, iris, conjunctivae, and chemosis. The individual mean score of opacity, iris, conjunctivae, and chemosis for animal no. 01 was 0.00, 0.00, 0.33, and 0.00, for animal no. 02 was 0.00, 0.00, 0.66, and 0.00, and for animal no. 03 was 0.00, 0.00, 0.66, and 0.00, respectively, while the individual mean score of opacity, iris, conjunctivae, and chemosis for the positive control is 2.33, 2.00, 0.00, and 0.00 respectively. Only one rabbit was used as it showed severe irritation reaction.

The rabbits were sacrificed after 21 days showing reversibility of ocular lesions after 72 hours.

Since the animals treated with extract showed the irritation score of ≤0.55 the outcome of result did not qualify for category 1 and 2 of the classification criteria. Hence aqueous extract of *Achyranthes aspera* is classified as per Harmonised Integrated Classification System (14 August, 2001) as “Not Irritating” to the rabbit eye.

## 4. Conclusion

This study showed good correlation between results obtained by the HET-CAM test and those of the acute eye irritation tests. From the present study, we conclude that aqueous extract of *A. aspera* is nonirritant and does not have any eye irritation potential.

## Figures and Tables

**Table 1:** Irritation score, severity, and effect classification in the *in vitro* HET-CAM assay.

Compound	Irritation score (mean)	Irritation severity (mean)	Classification of the effect
0.9% NaCl Negative control	0.07	0	No reaction
1% SDS Positive control	12.80	3	Severe reaction
0.1 N NaOH SDS Positive control	14.84	3	Severe reaction
*A. aspera *aqueous extract	0.07	0	No reaction

**Table 2:** Grading of ocular lesions by acute eye irritation test in rabbits.

Animal numbers	01				02				03				Mean	04				Mean
At hours	1	24	48	72	1	24	48	72	1	24	48	72		1	24	48	72	
Reactions	Aqueous extract of *Achyranthes aspera *													Positive control (1% SDS)				
Corneal opacity	0	0	0	0	0	0	0	0	0	0	0	0	0	2	3	2	2	2.33
Area of opacity	0	0	0	0	0	0	0	0	0	0	0	0	0	3	2	2	2	2.00
Iris	0	0	0	0	0	0	0	0	0	0	0	0	0	0	0	0	0	0
Conjunctivae	1	1	0	0	1	1	1	0	1	1	1	0	0.55	0	0	0	0	0
Chemosis	0	0	0	0	0	0	0	0	0	0	0	0	0	0	0	0	0	0

Note: grading of area of cornea involved.

Zero: 0.

One quarter (or less) but not zero: 1.

Greater than one quarter, but less than half: 2.

Greater than half, but less than three quarters: 3.

Greater than three quarters, up to whole area: 4.

## References

[2] Tavaszi J, Budai P (2007). The use of HET-CAM test in detecting the ocular irritation. *Communications in Agricultural and Applied Biological Sciences*.

[3] Eskes C, Bessou S, Bruner L (2005). Eye irritation. *Association of Trial Lawyers of America*.

[4] Kishore S, Surekha PA, Sekhar PVR, Srinivas A, Balakrishna P (2008). Hen egg chorioallantoic membrane bioassay: an in vitro alternative to draize eye irritation test for pesticide screening. *International Journal of Toxicology*.

[5] Srivastav S (2011). *Achyranthes aspera*—an important medicinal plant: a review. *Journal of Natural Product and Plant Resources*.

[6] Girach RD, Khan ASA (1992). Ethnomedicinal uses of *Achyranthes aspera* leaves in Orissa (India). *International Journal of Pharmacognosy*.

[7] Bkher L, Haensel R, Keller K, Rimpler G, Schneider G (1992). Achyranthes. *Hagers Handbuch der Pharmazeutischen Praxis*.

[8] ICCVAM-Recommended Test Method Protocol (2010). Hen’s egg test—chorioallantoic membrane (HET-CAM) test method.

[9] Dehelean CA, Alexa E, Feflea Ş, Pop G, Peev C (2011). Ochratoxin a: a toxicologic evaluation using in vitro and in vivo bioassays. *Analele Universităţii din Oradea, Fascicula Biologie*.

[10] OECD (2001). *Harmonised Integrated Classification System for Human Health and Environmental Hazards of Chemical Substances and Mixtures*.

